# Removal of GIT lesions and the role of impedance of the injection solution—an innovative approach to known methods

**DOI:** 10.1007/s00109-024-02457-1

**Published:** 2024-06-03

**Authors:** Martina Lösle, K. E. Grund, B. Duckworth-Mothes

**Affiliations:** 1 Institute of Laboratory Animal Science, University Zurich, Wagistr. 12, 8952 Schlieren, Zurich, Switzerland; 2 Department of General, Visceral and Transplant Surgery, Experimental Surgical Endoscopy, University Hospital Tübingen, Tübingen, Germany; 3 Current Affiliation: Experimental Endoscopy, Development and Training, Internal Medicine I - Gastroenterology, Gastrointestinal Oncology, Hepatology, Infectiology and Geriatric Medicine, University Hospital Tübingen, Tübingen, Germany

**Keywords:** EMR, ESD, Impedance, GIT lesions, Polypectomy, Injection solution

## Abstract

**Abstract:**

In this work, for the first time, the specific impedances of various injection solutions as well as the surface and tissue impedance after injection of these solutions were analyzed and compared regarding the radio-frequency surgical cutting process. The impedances of 0.9% NaCl, 4% gelatine, 6% hydroxyethyl starch, 10% glycerol/5% fructose, 10% glucose, 5% and 20% albumin, blood, and blood plasma as well as aqua destillata have been tested in vitro. Even if EMR and ESD are routinely used in clinical practice, there is so far no easy, fast, and safe method to remove larger lesions *en bloc.* We show that the impedance of the injected solution shows to be a crucial factor for safe removal, especially of larger lesions (Ø > 20 mm) and more importantly in accordance with the requirements of oncology and pathology.

**Key messages:**

- Impedance is playing a crucial factor in the radio-frequency (RF)-surgery.
- With a higher Impedance there will be less current necessary to reach the aimed voltage.
- Injection solution Aqua destillata and 10% Glucose, show significantly higher Impedances.
- Higher impedances lead to less surgical related complications.
- Minor changes in existing method to improve patent safety.

## Background and study aims

Endoscopic mucosal resection (EMR) is one of today’s routine methods to remove gastrointestinal lesions and shows good results for lesions Ø ≤ 2 cm [1]. In this size, a removal of the lesion *en bloc* and *in sano* is achieved and therefore fulfills the criteria of oncology and pathology.

The removal of larger (Ø > 20 mm) and more difficult lesions however is still a challenge for radio-frequency (RF) surgery.

Larger lesions can often only be removed with EMR via *piece-meal* technique which leads to higher rates of complications and a higher risk of recurrence [1, 2]. Therefore, the method of endoscopic submucosal dissection (ESD) is often used in these cases, allows the removal *en bloc*, and reduces the rates of recurrence. However, the technique of ESD shows significantly higher rates of complications like bleeding and perforations; it is more expensive and it is a challenge to learn and train [3].

Both methods, EMR and ESD, need 200 V to perform a clean RF surgical cut. In accordance with Ohm’s law (*U* = R*I), more intensive surface contact of the surgical instrument resp. snare leads to a decreased resistance (*R*)/impedance, and hence to delayed cutting. If this voltage peak of 200 V is not exceeded, the cutting process will not start, and most energy will be transformed to heat and results in thermal necrosis and secondary perforation. This is where tissue impedance (the vector combination of resistance and reactance) plays a critical role. According to Ohm’s law, the necessary voltage can only be achieved if there is a relatively high tissue impedance because the current, supplied by the RF generator, is always—independent on the manufacturer—limited to maximum of 1.5–2 A [4].

To avoid a perforation and/or thermal devitalization of surrounding tissue, the lesion to be removed is often elevated by injection of physiological saline solution (0.9% NaCl). During the surgical process, this can be repeated and adjusted. There are several studies analyzing various injection solutions regarding the height and duration of this elevation [5–8]; however, the interactions between the submucosal injection and the RF parameters, especially the impedance, are not considered.

Previously done experiments strongly suggest that an adapted impedance of the tissue is essential to fulfill the requirements of pathological and oncological assessments.

Our aim was to find an optimal submucosal injection solution to allow adapted impedances in tissue and therefore to enable the removal of larger lesions. Adapted impedances enable the necessary voltages and consequently reduce the complication rates.

## Methods

The following clinically relevant submucosal injection solutions have been tested:

- 0.9% saline solution (standard reference solution) (Fresenius Kabi, Bad Homburg, Germany)
- Gelafundin (4% gelatine) (B. Braun, Melsungen, Germany)
- 6% Hydroxyethyl starch (HES 20.5) (Fresenius Kabi)
- Glucosteril (10% glucose in aqua destillata) (Fresenius Kabi)
- A mix of 10% glycerol/5% fructose in 0.9% saline solution (self-made)

Additional analyses regarding the impedance have been conducted with 5% respective 20% albumin as well as with autologous human blood.

With standardized gold electrodes (1 mm/15 mm) and with a RF impedance meter (*f* = 100 kHz), the specific impedances and tissue impedances with the different solutions were measured.

The volume of 3 ml of each solution was injected in the submucosa of isolated porcine stomach (corpus and fundus area). The whole procedure was done within physiological relevant temperature of approx. Ø 37 °C. The impedance was measured at the surface as well as in the tissue. This realistic in vitro model allowed to determine not only the impedance but also the height and duration of the elevation.

Additionally, the height of the elevation was analyzed computer-based (ImageJ, Wayne Rasband, NIH, USA) after the injection of 2 ml of each solution for 30 min into fresh tissue.

Statistics were done using one-way ANOVA in GraphPad (GraphPad Software, San Diego, USA).

## Results

**Fig. 1:**
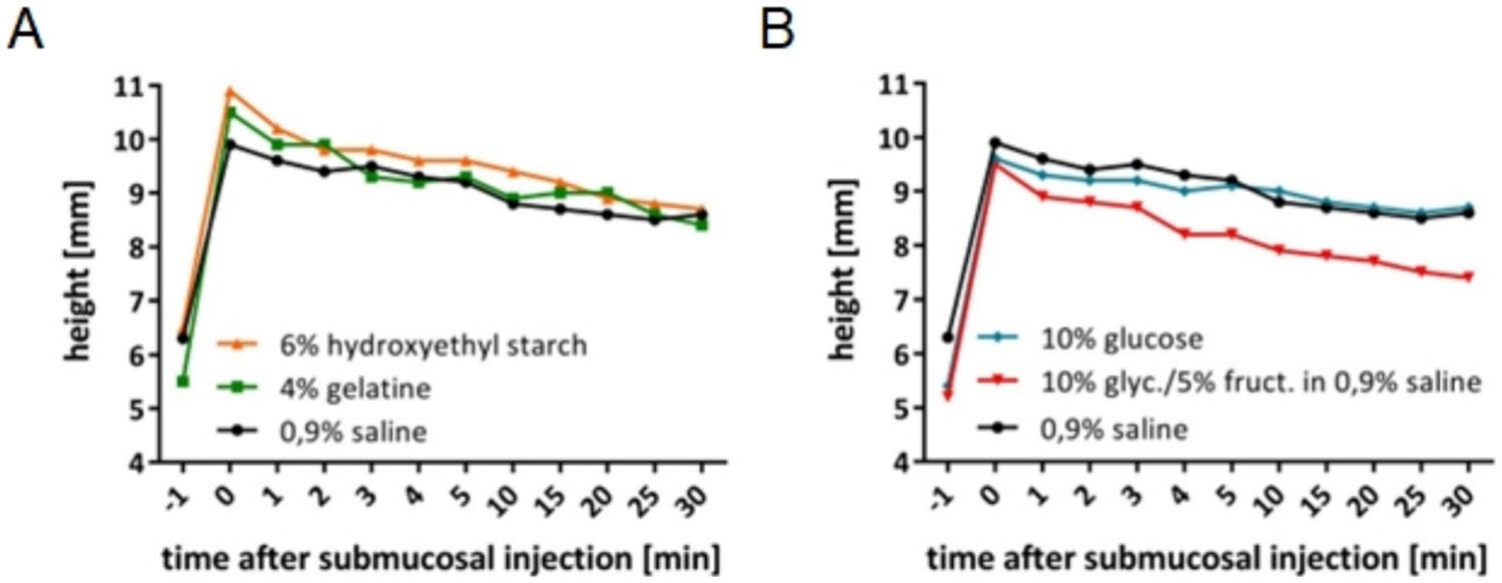
Height of elevation during a defined time period. Measured after injection of 2 ml of each solution over a time period of 30 min. In this graph, − 1 represents the tissue at starting point, and 0 represents the timepoint directly after injection into the submucosa. Shown are the medians of each timepoint (*n* = 5). For better overview separated into two graphs

In a clinical setting, the choice of the injection solution is mostly determined by the height and duration of the elevation. Therefore, we measured the height of the elevation over a defined period of time, comparing the solutions which are commonly applied in clinical use with each other (Fig. 1).

Analyzing the height of the elevation during 30 min after injection, no significant difference between the various solutions could be detected.

**Fig. 2:**
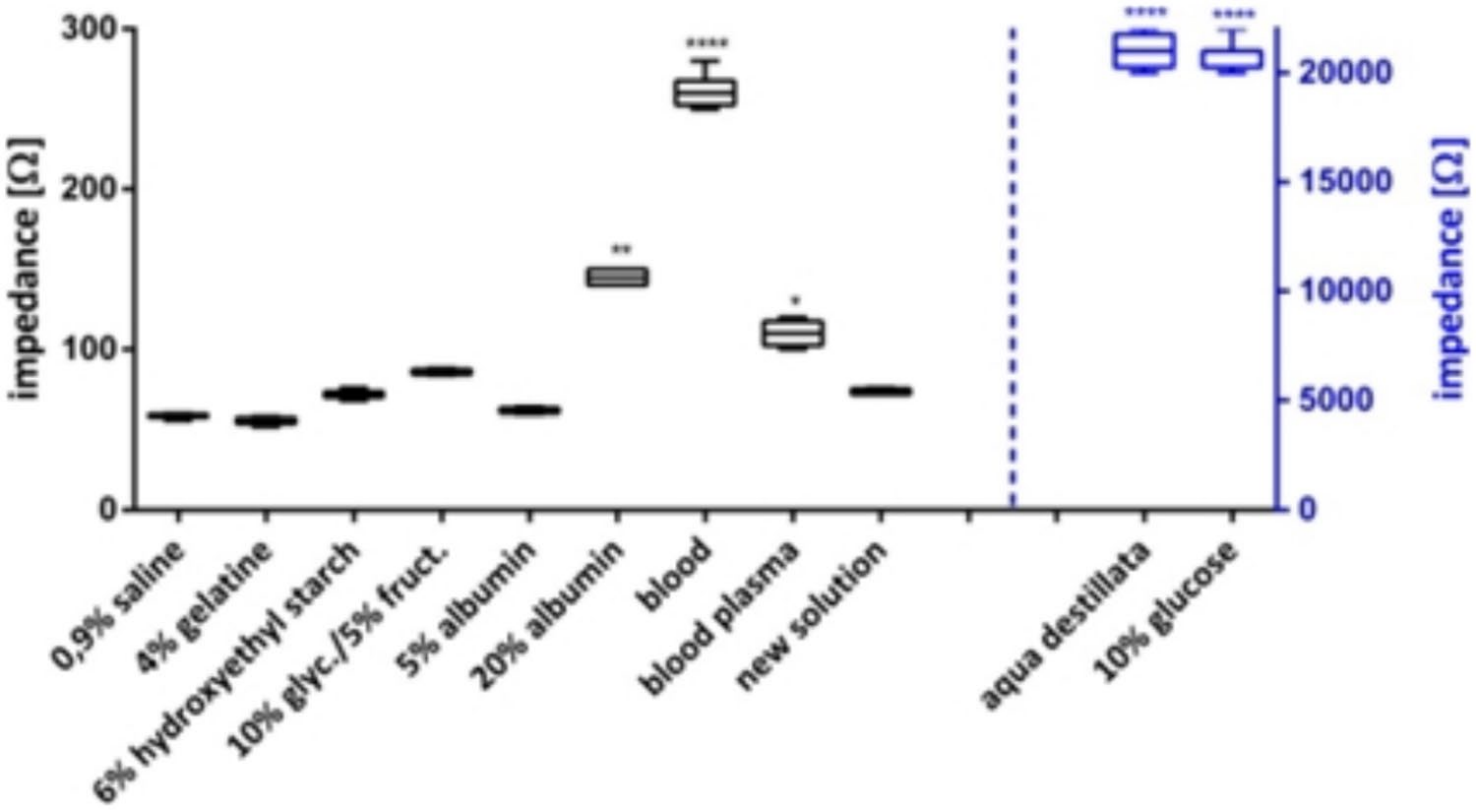
Specific impedances from various submucosal injection solutions. Significance in comparison to 0.9% NaCl solution. The graph shows range and quartiles, *n* = 5. *p ≤ 0.05, **p ≤ 0.01, and ***p ≤ 0.001, ****p ≤ 0.0001

Subsequently, the specific impedance from each solution per se was tested. All solutions are used in the clinical daily routine (0.9% NaCl, 4% gelatine, 6% hydroxyethyl starch, 10% glycerol/5% fructose, 10% glucose). Additionally, we used solutions that are discussed in literature as a possible alternatives (5–8) (5% and 20% albumin, blood, and blood plasma) as well as aqua destillata and a new anonymized injection solution (Fig. 2).

Aqua destillata and 10% glucose solution show a significantly higher specific impedance (*p* < 0.0001) compared to the other tested solutions. Both show a 400 times higher impedance than the 0.9% NaCl solution. Also 20% albumin, blood plasma, and especially full blood show significantly higher impedance compared to the standard solution 0.9% NaCl.

**Fig. 3:**
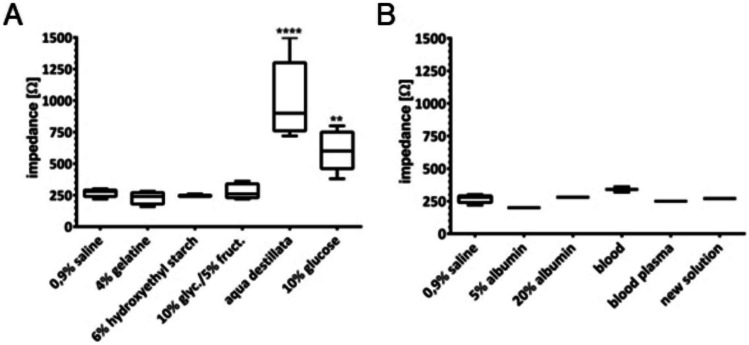
Surface impedance. Measured at 37 °C after injection of 3 ml into the submucosa. Significances in comparison to standard solution 0.9% NaCl. Shown are range and quartiles, **A** *n* = 5; **B** *n* = 2. *p ≤ 0.05, **p ≤ 0.01 and ***p ≤ 0.001, ****p ≤ 0.0001

**Fig. 4:**
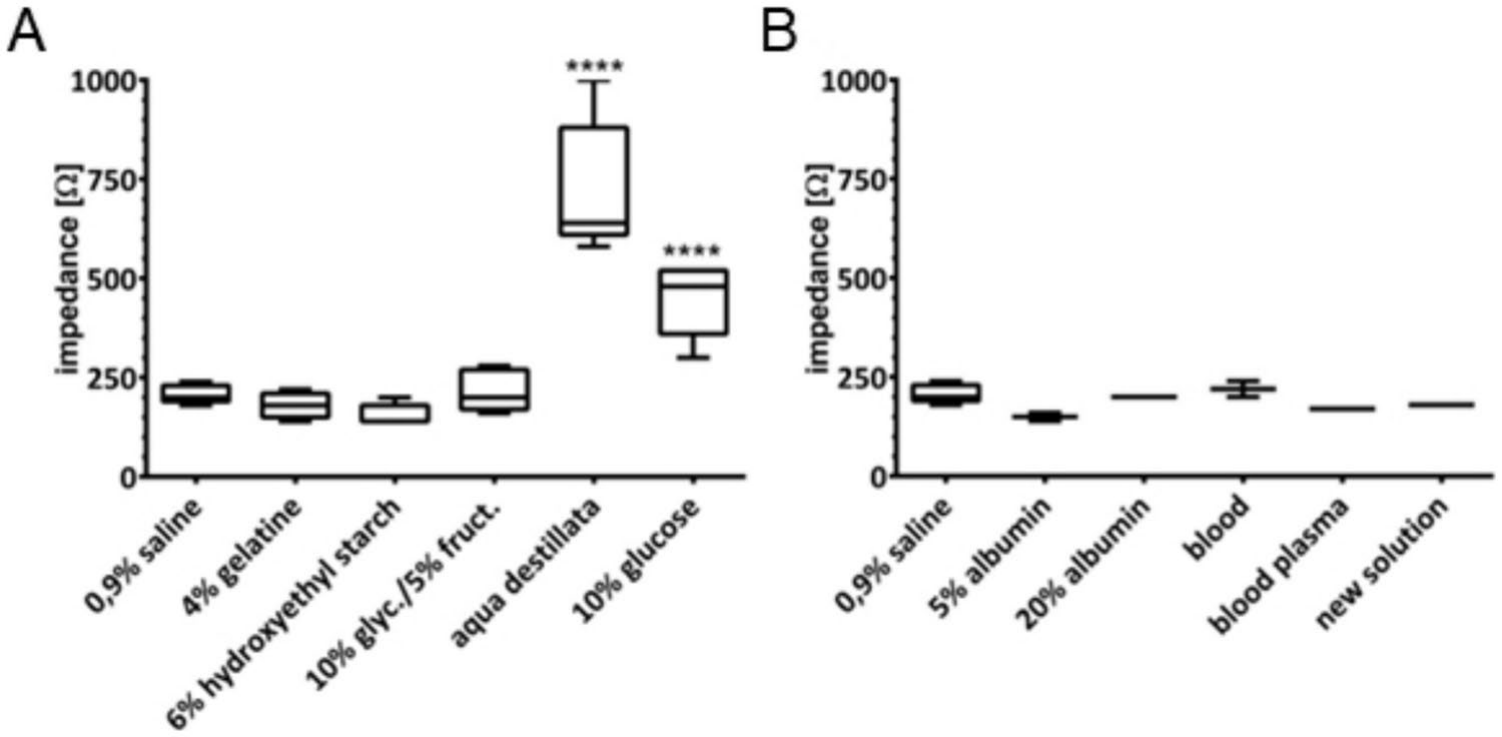
Impedances inside the tissue. Measured at 37 °C after injection of 3 ml each into the submucosa. Significances compared to 0.9% NaCl solution. Graph shows range and quartiles, **A** *n* = 5; **B** *n* = 2. *p ≤ 0.05, **p ≤ 0.01 and ***p ≤ 0.001, ****p ≤ 0.0001

All of the injection solutions have then been subsequently tested for their impedance after injection of 3 ml each. The impedance was measured at the surface (Fig. 3) and within the tissue (Fig. 4) of the fundus area.

After injection into the tissue, 10% glucose and aqua destillata still show higher impedances at the surface (Factor 3,7 respective 2,3). The other tested solutions show no significant difference compared to standard solution 0.9% NaCl.

## Measurements inside the submucosa

Aqua destillata and 10% glucose show the highest impedance (Factor 3,5 respective 2,2). The other tested solutions show no significant difference regarding tissue impedance compared to 0.9% NaCl solution.

## Discussion

According to the Guidelines (S3-Leitlinie Kolorektales Karzinom 2019), the colorectal carcinoma is with an incidence of more than 64,000 and a death toll of 26,000 a year the prevalent malignant tumor in Germany [9]. Colonoscopy is by far the most sensitive and specific method to detect colorectal neoplasia and therefore is recommended as a gold standard.

Removal of polyps before they develop to a carcinoma can significantly reduce the rates of colorectal carcinoma [10]; however, there is a misrate in lesions which are not detected within colonoscopy (5%) in high-risk patients up to 66%. Often, it is the flat polyps and adenomas which are missed. That is why Jung et al*.* point out not only the importance of follow-up colonoscopies but also the necessity of technical improvements.

For the esophagus, there are studies that directly compare the methods of EMR and ESD in the removal of *superficial esophageal cancer* (SEC). It shows a higher success rate in the removal of lesions < 2 cm regarding the *en bloc* resection as well as the curative resection rate for ESD. However, ESD takes much more time than EMR and has a higher risk potential. Comparing the rate of bleedings, no difference is seen; however, the ESD shows a higher rate of perforations [11].

While there are promising developments on the technical side of flexible endoscopy like new resection instruments [12, 13], we think that the results of resection can be improved by choosing an optimized injection solution.

The commonly used injection solution is 0.9% NaCl. That is why in most studies 0.9% NaCl is used as standard injection solution. Studies show that 0.9% NaCl gives a good initial elevation which then declines relatively fast compared to, e.g., 10% glycerin/5% fructose solution [5, 7]. In vitro a longer lasting elevation of 10% glycerin/5% fructose solution compared to 0.9% NaCl is shown in human tissue [7] but cannot be seen in porcine tissue (1 ml) [5] and the effect of better results for an *en bloc* resection is limited to lesions < 2 cm. The same holds true for most tested glucose solutions; however, due to the high osmolarity of these solutions (glucose > 15%), tissue damages can be observed [5, 7]. This is why in this study glucose solutions of 6% have been used.

Other studies used the Hydro-Jet technique to inject 5 ml 4% gelatine, 10% hydroxyethl starch, and 50% glucose solution compared to 0.9% NaCl in vivo in pigs that showed a higher elevation and a slower decline for hydroxyethyl starch and glucose solution. A snare-resection afterwards was performed without problems [6].

Our work shows no significant difference in height and duration of the elevation within all tested solutions. These varying findings show the dependence of the results from the kind of tissue, technique, and volume of injection solution that is used.

There are other studies that discuss the usage of autogenic blood as injection solution. Compared to 0.9% NaCl, it shows higher and longer lasting elevations; it is available and very cost effective, but it can lead to blood coagulation and impaired sight during the procedure [8, 14].

In this work, for the first time, the specific impedances of surface and tissue after injection of various solutions were analyzed and compared regarding the laws of every RF surgical cutting process. It clearly shows that aqua destillata and 10% glucose, to a lesser content also autogenic blood, lead to significantly higher impedances and therefore can optimize the electrosurgical cutting results. These results should be respected in further research in RF surgery as well as in clinical practice, especially for larger lesions or in critical situations. If a problem with the cut occurs during a procedure a low-impedance solution could be injected additionally to 0.9% saline solution. Other tested solutions did not show any significant differences regarding their impedance and are at the same level than the standard solution.

In accordance to our hypothesis, these solutions did not show any better results in the cutting process at lesions > 2 cm (data not shown).

Our findings underscore the significance of comprehensively understanding the electrosurgical cutting process, particularly in critical cases, and emphasize the role of impedance as a crucial factor for achieving the necessary voltage for cutting. Further research in this area is imperative for enhancing safety and reducing the risk of bleeding or even perforation. We think our research illuminates the potential for innovation in conventional resection techniques like polypectomy and EMR, as well as in advanced methods such as ESD and ESR. Prioritizing impedance considerations could be the beginning of a new field of research, one that merits attention not only in research but also in everyday clinical practice, by ensuring safety for patients by mitigating risks of RF surgery.

## Data Availability

The datasets generated during and/or analyzed during the current study are not publicly available due to being basic research and not part in any registered study. It is available from the corresponding author on reasonable request_._
